# Comparison of root morphology and rhizosphere microbial communities form moso‐bamboo in different forest types

**DOI:** 10.1002/ece3.10153

**Published:** 2023-06-06

**Authors:** Jingyu Liu, Huixuan Liao, Minghua Fan, Ting Zhou, Shaolin Peng

**Affiliations:** ^1^ State Key Laboratory of Biocontrol, School of Life Sciences Sun Yat‐sen University Guangzhou China

**Keywords:** bamboo, broadleaf forest, coniferous forest, microbe, root morphology, underground mechanism

## Abstract

Moso‐bamboo (*Phyllostachys edulis*), with the favor of human disturbance, rapidly invades adjacent forests to form monocultures in East Asia. Moso‐bamboo not only intrudes the broadleaf forests but also the coniferous, and it could impact by above‐ and below‐ground pathways. However, it still remains unclear whether the below‐ground performance of moso‐bamboo differs from broadleaf to coniferous forests, especially those differing in competitive and nutrient acquisition strategies. In this study, we investigated three types of forest stands in Guangdong, China, including a bamboo monoculture, a coniferous forest, and a broadleaf forest. We found that moso‐bamboo may suffer stronger soil P limitation (soil N/P = 18.16) and may be infected by more AMF in coniferous than broadleaf forests (soil N/P = 16.17). Based on our PLS‐path model analysis, soil P resource may be the key to differ moso‐bamboo root morphology and rhizosphere microbe in different forests: in broadleaf forests with weaker soil P limitation, may be realized through increasing specific root length and specific surface area, whereas in coniferous forests with stronger soil, P limitation may be realized through combining more AMF. Our study highlights the importance of underground mechanisms about moso‐bamboo expansion in different forest communities.

## Graphical abstract

We compared the rhizosphere soil properties, rhizosphere microbial abundance, and root morphology differences of moso‐bamboo in three types of forest stands. Our result suggests that moso‐bamboo regulates its root morphology and rhizosphere microbial abundance with the soil property changes in different types of forest stands; besides, moso‐bamboo neither relies on soil P supply nor the combination with the rhizosphere microbe. Our work could provide new ideas for forest management to control moso‐bamboo invasion.

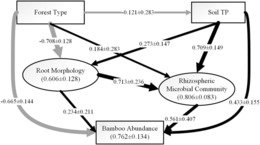

## 1 INTRODUCTION

Bamboo intruding into adjacent forests and making huge damage has become a global ecological issue, especially in Eastern Asia (Fukushima et al., 2015; Li et al., 2019; Liu et al., 2019; Wang, Bai, et al., 2016; Wang, Sasaki, et al., 2016). Moso‐bamboo (*Phyllostachys edulis*), a widely cultivated tree‐like clonal plant in southern China, expands very rapidly, and the area of moso‐bamboo forests increased by 14.5% from 2004 to 2013 in this area (Xu et al., 2020). The invasion of moso‐bamboo has caused many problems in local ecosystems, such as biodiversity loss, forest landscape destruction, and soil degradation (Larpkern et al., 2011; Okutomi et al., 1996). Thus, the mechanisms of moso‐bamboo expansion have received much attention.

Moso‐bamboo can inhibit the growth of forest trees through light competition and physical damage to roots and shoots (Griscom & Ashton, 2003). Although above‐ground mechanisms for bamboo invasion made a more intuitive impact, below‐ground mechanisms have been suggested to play a more important role in dictating bamboo expansion compared with above‐ground mechanisms (Lin et al., 2014; Qin et al., 2017). Moso‐bamboo expansion can change soil physiochemical properties and the soil microbial community of the original forests (Wang, Sasaki, et al., 2016), some studies indicated that soil pH, organic C, N, and P contents were reduced after moso‐bamboo invasion (Fukushima et al., 2015), while limiting the C and N uptake by neighboring trees (Wang, Sasaki, et al., 2016). Since soil P is often limited in areas where moso‐bamboo is common (Li et al., 2019), soil P is also an important factor for moso‐bamboo expansion, which is likely to affect bamboo invasion interactively with rhizosphere microbial community and root morphology. In addition, many studies have suggested that the rhizosphere microbial community of moso‐bamboo is altered significantly during it intruding into adjacent forests (Wang et al., 2017; Wang, Sasaki, et al., 2016; Wang, Tian, & Chiu, 2016; Xu et al., 2020), which can increase the total abundance of soil microbes and the relative abundance of bacteria versus fungi (Lin et al., 2014; Tripathi et al., 2005).

So far, mechanistic studies on moso‐bamboo expansion mostly focused on broadleaf forests (Fukushima et al., 2015; Wang, Tian, & Chiu, 2016; Xu et al., 2020). However, moso‐bamboo can also intrude into coniferous forests (Komatsu et al., 2010; Wang, Bai, et al., 2016). Studies on the root plasticity of moso‐bamboo during expansion into conifer‐broadleaf mixed forests have shown that the density, length, and rate of fine roots of bamboo increase (Cai et al., 2019). Although previous evidence indicated that moso‐bamboo invasion in coniferous forests may differ in underground processes from those in broadleaf forests (Liu et al., 2019), no studies have compared the different performances of moso‐bamboo across forest types. In this study, we tried to answer two questions: (1) are there differences in the rhizosphere microbial community and root morphology of moso‐bamboo across forest types? (2) how do such differences correlate to dominating plant change? Moso‐bamboo expansion through altered rhizosphere microbial community and root morphology could shed light on how important underground mechanisms are in different forests, potentially allowing better management of bamboo forests to conserve broadleaf/coniferous forests and make good use of bamboo resources.

## 2 MATERIALS AND METHODS

### 2.1 Study site

The study was conducted in forest areas in the Nankunshan Natural Reserve, Guangdong Province, China (23°30’N, 114°38′ E; 504 ~ 561 m a.s.l.). This area is in a subtropical monsoon climate region with an annual mean air temperature of 23°C and annual average precipitation of 2163 mm. The difference is obvious between the dry and wet seasons, and precipitation is mostly concentrated from April to September. Moso‐bamboo mainly expanded by natural diffusion locally in the last century. Artificial coniferous forests (especially the Chinese fir forest) are widely distributed in this area and are invaded by moso‐bamboo with high abundance in composition (detailed location, see Figure S1). Moreover, based on the vegetation data, the invasion in coniferous forests is more serious than that in local broadleaf forests, the importance value of moso‐bamboo was more than twice of the Chinese fir in the coniferous forests (Appendix S1).

In 2016, the experimental sites were selected in three different types of forests (moso‐bamboo forest, adjacent coniferous forest, and broadleaf forest), based on the classification of the forest type depending on the dominant species (Appendix S1). Six plots (10 × 10 m) were established at each site.

### 2.2 Soil sampling and analysis

In each plot, soil samples were collected after cleaning the litter on the ground of the sampling points. Mineral soil samples of the 0–15‐cm layer (under the humus layer) were obtained using a stainless‐steel cylinder (diameter = 7.0 cm, height = 15.0 cm) at each point. Soil samples from the same plot were mixed together equably. Because one sample of the coniferous forest was lost when it was transported back to the laboratory, and because each plot needed to include moso‐bamboo, one broadleaf forest plot was not sampled because of the absence of moso‐bamboo. Thus, the total number of soil samples was 16 (6 samples in bamboo forest and 5 samples in both coniferous forest and broadleaf forest). After the soil samples were taken back to the laboratory, they were air‐dried. The dry soil samples were sieved by a 2‐mm‐mesh sieve to remove the gravel and plant tissue for soil characteristic analysis. Soil pH (H_2_O) (air‐dried soil, H_2_O 1:5 w/v) was measured using a pH meter fitted with a glass electrode. Total C (TC) was measured with the high‐temperature external thermal potassium dichromate oxidation‐volumetric method, total N (TN) was measured with the sulfuric acid‐hydrogen peroxide boiling‐distillation titration method, and alkaline nitrogen (AN) and total P (TP) contents were measured with the sulfuric acid‐hydrogen peroxide boiling‐vanadium molybdenum yellow colorimetric method.

### 2.3 Bamboo root morphology analysis

Moso‐bamboo was selected in each plot to collect root and soil samples, which were acquired at two points on the stem base of each bamboo plant by stainless‐steel cylinder (diameter = 7.0 cm, height = 15.0 cm) boring. The samples were placed in plastic bags and transported back to the laboratory. The roots and soil were separated by sieving and tweezers to collect soil samples adhering to the roots. After separation, the root samples were washed and dried at 70°C for 48 h before root morphology analysis, and the soil samples from the same bamboo were blended and packaged to store at 4°C briefly for phospholipid fatty acid (PLFA) analysis.

The root mass was measured by an electric scale, and the total root length (L), root superficial area (SA), root average diameter (AD), and fine root (diameter <2 mm) length were measured using root analysis system scanning. The other indicators were calculated using the following equations (Bolte & Villanueva, 2005):

(1) $$B=w/\left[\pi \times \left(d/2\right)\;2\right]$$

(2) SRL=L/w

(3) SSA=SA/w

(4) RLD=B×SRL

Here, B is root biomass (g/cm^2^); w is root mass (g); d is inner diameter of the cylinder (cm); L is height of the cylinder (cm); SRL is specific root length (cm/g); SSA is specific superficial area (cm^2^/g); and RLD is root length density.

### 2.4 Mycorrhizal infection rate of bamboo's root

The bamboo roots were cleaned and cut 1–2 cm sections of the root tip. The root samples were softened in 20% KOH with 121°C heating, then, bleached in alkaline hydrogen peroxide (10% H_2_O_2_ and 25% NH_3_·H_2_O) and acidized in 5% acetic acid. After pre‐treatment, the samples were stained with 0.05% trypan blue. To estimate the percentage of mycorrhizal colonization of roots, we used the magnified intersections method (McGonigle et al., 1990).

### 2.5 Microbial biomass and community structure

Soil microbial biomass and community composition were examined using PLFA analysis. PLFAs are the major components of the membranes of living cells, so the amount and composition of PLFAs in soils have been used as an index of the total microbial biomass and as a fingerprint of the microbial community structure, respectively (Frostegård et al., 2011).

The rhizosphere soil samples for PLFA measurement were stored at 4°C and treated immediately. An aliquot of 8 g (dry weight) of soil was extracted twice using a chloroform–methanol‐citrate buffer mixture (6:12:5 by volume) in a centrifuge tube. Phosphoric acid buffer and chloroform were added every 12 mL to the supernatant to oscillate and then poured into a separator funnel to stand overnight. The next day, the chloroform phase obtained from the lower funnel was bathed at 30°C and blow‐dried with high‐purity nitrogen. The methanol phase was collected using a hyperactive silica gel column and then blow‐dried with high‐purity nitrogen. Methanol‐toluene (1:1 by volume) and 0.2 mol/L KOH methanol solution were added to each 1 mL sample and incubated at 37°C for 15 min. Two milliliters of pure water, 0.3 mL of glacial acetic acid, and 2 mL of n‐hexane (Kermio) were added, swirled, and mixed for 30 s to extract FAMEs in the upper layer later. In this experiment, 19:0 was used as an internal standard. The PLFAs were then purified from the lipid extracts, quantified, and identified using a gas chromatograph. The total content of 7 major PLFAs (total PLFAs) was used as an indicator of total microbial biomass in the soil sample (Frostegård et al., 2011; Vestal & White, 1989; Zelles, 1999).

### 2.6 Statistical analysis

We used one‐way ANOVA to analyze the effect of forest type including pure bamboo forest, coniferous forest, and broadleaf forest on soil properties, PLFA contents, and bamboo root morphology, and clarify the difference in microbial community composition. To test whether the soil P, rhizosphere microbial community, and root morphology account for moso‐bamboo expansion, we conducted a partial least square (PLS‐path) path model to detect all significant direct and indirect correlations among the forest vegetation types, soil P and moso‐bamboo abundance (Table S1) using the “plsm” function from the “plsm” package. The measurements of soil P content, rhizosphere microbial PLFA contents, and bamboo root morphology were calculated for each individual. The data were statistically analyzed by SPSS 19.0 and R 3.3.1.

## 3 RESULTS

### 3.1 Soil C, N, and P contents

**FIGURE 1:**
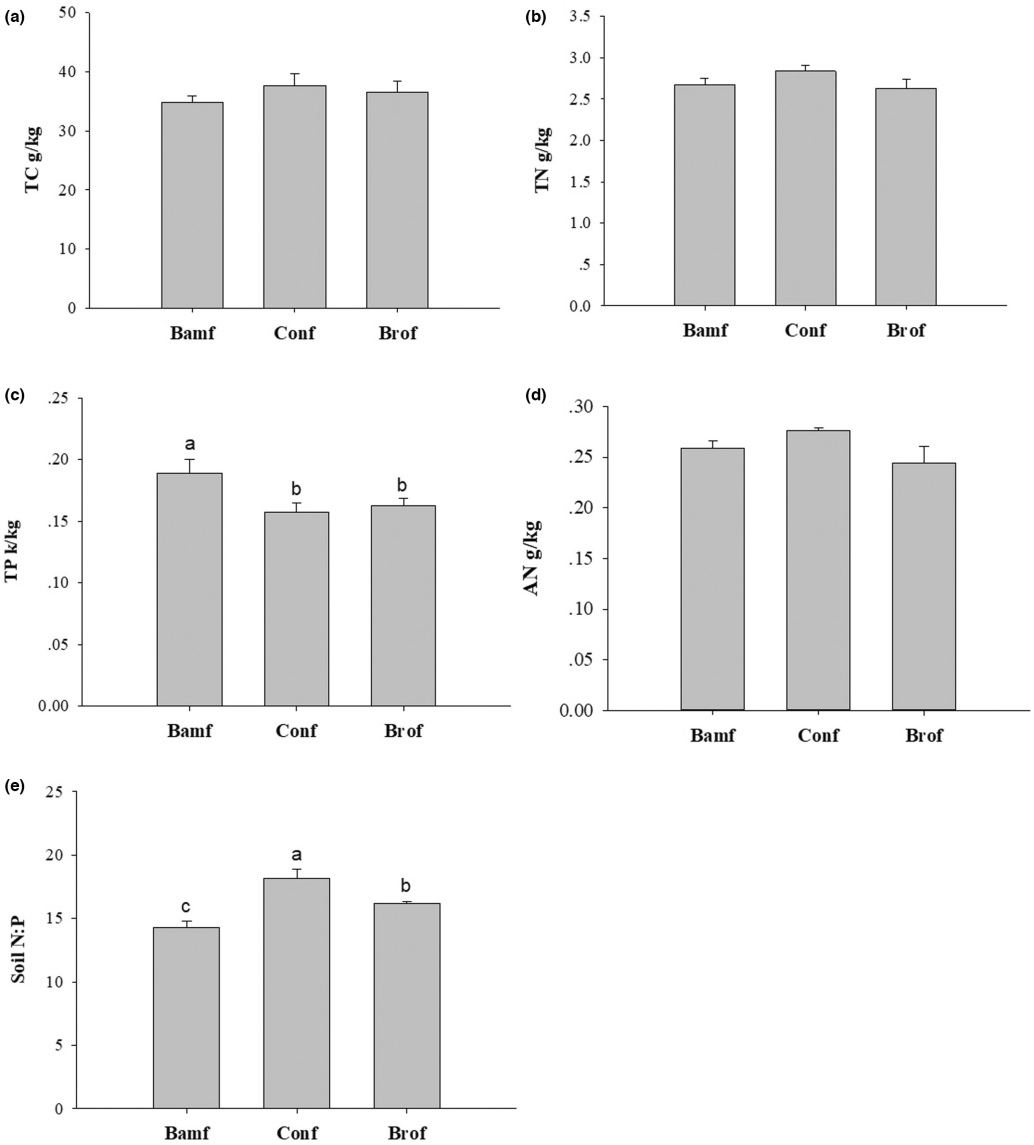
Differences in soil properties between different forest stands based on Tukey's LSD test. Bamf, bamboo forest stand; Conf, coniferous forests stand; Brof, broadleaf forest stand. Significant differences are indicated by different letters (*p* < .05).

Background soil in our study area was a typical red soil with low pH and low nutrient content (Figure 1). There was no significant difference in either soil C or N between the three forest stands (i.e., bamboo, coniferous, and broadleaf forests), while the soil P in the bamboo forest was significantly higher than that in the other two forests (Figure 1a–c). The soil N:P was also significantly different among these stands, which was highest in the coniferous stand, while it was the lowest in the bamboo stand (Figure 1e).

### 3.2 Mycorrhizal infection rate of bamboo's root

**FIGURE 2:**
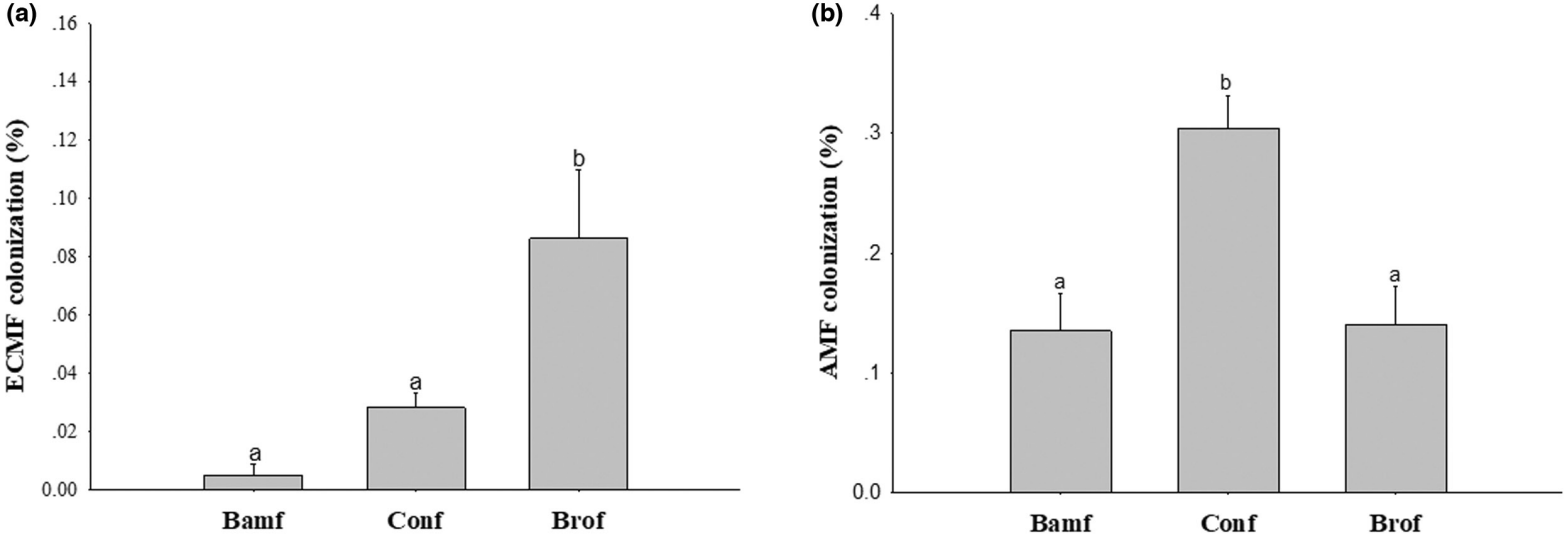
Differences in mycorrhiza colonization in moso‐bamboo between different forest stands based on Tukey's LSD test. Bamf, bamboo forest stand; Conf, coniferous forests stand; Brof, broadleaf forest stand. Significant differences are indicated by different letters (*p* < .05).

Roots of bamboo in the broadleaf forest had a significantly higher ECMF infection rate than the other two stands (Figure 2a), but all the infection rates were too low (<0.2%). As to AMF, the bamboo root in the coniferous forest stand was infected by more AMF than the pure bamboo and broadleaf forest stands (Figure 2b).

### 3.3 Bamboo rhizosphere microbial community composition

**FIGURE 3:**
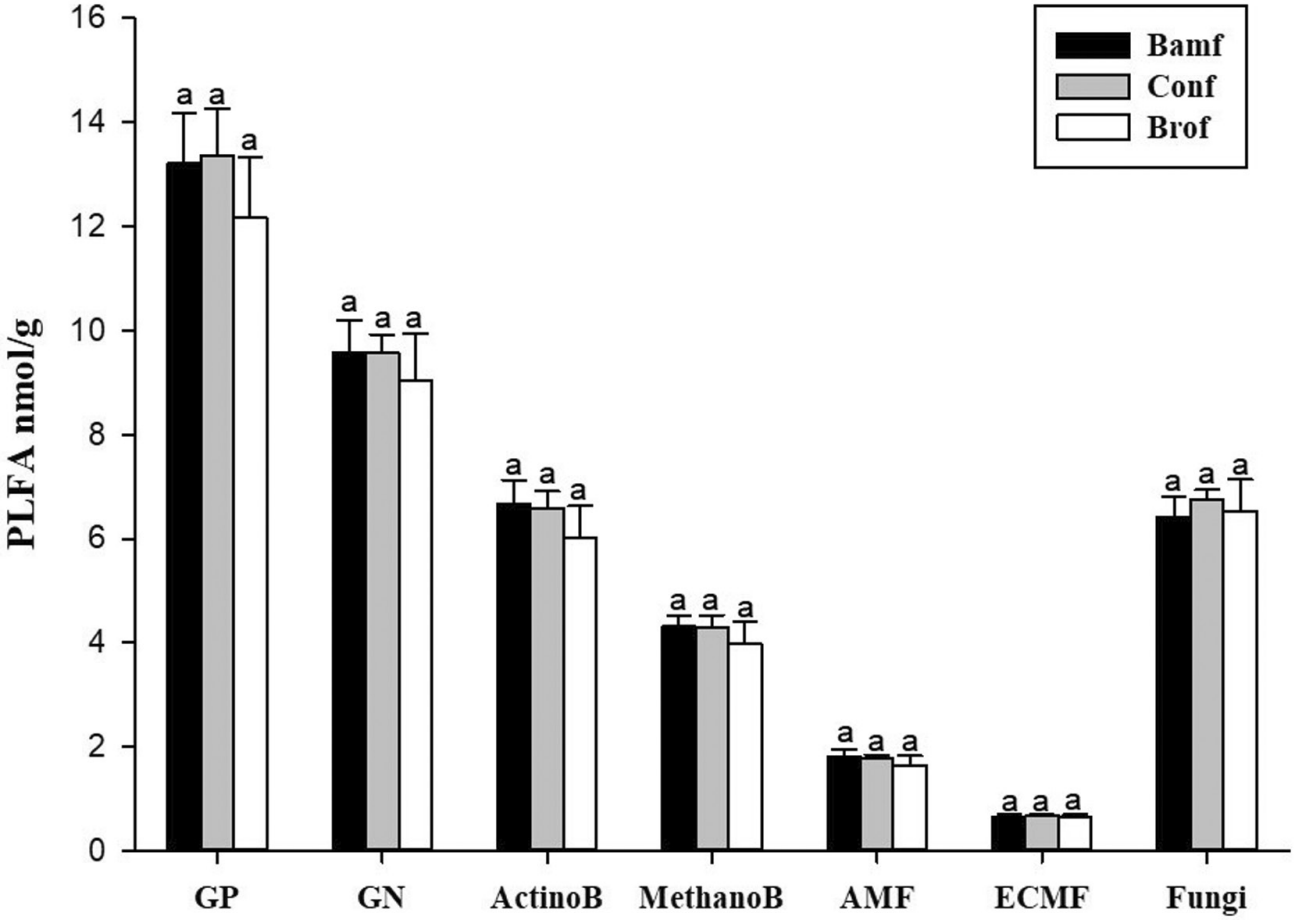
Differences in the abundance of major rhizospheric microbial groups of moso‐bamboo between different forest stands based on Tukey's LSD test. GP, Gram‐positive bacteria; GN, Gram‐negative bacteria; ActinoB, Actinobacteria; MethanoB, Methanotrophic bacteria; Fungi, Non‐AM/ECM fungi; Bamf, bamboo forest stand; Conf, coniferous forests stand; Brof, broadleaf forest stand. Significant differences are indicated by different letters (*p* < .05).

**FIGURE 4:**
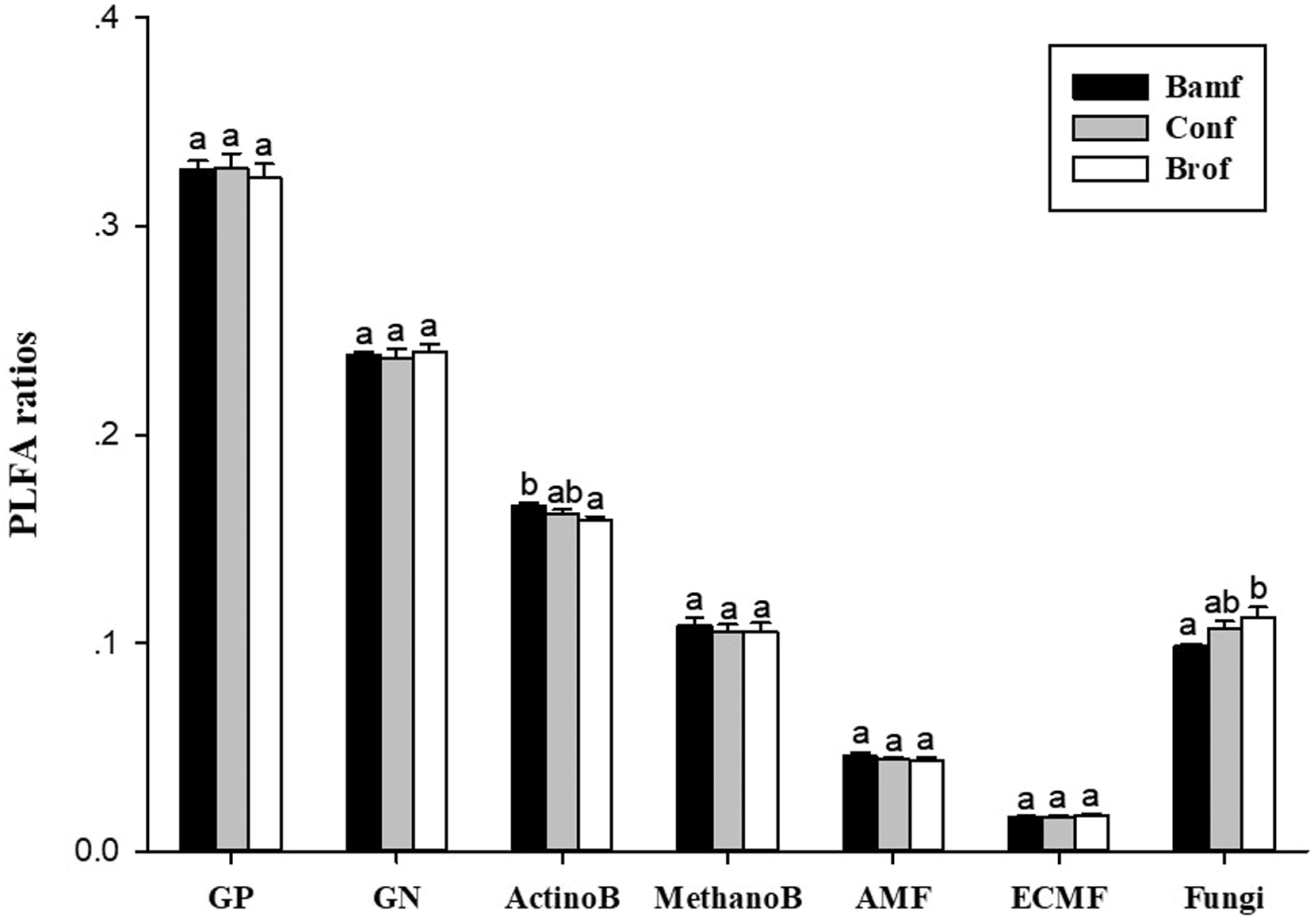
Differences in the relative proportions of major rhizospheric microbial groups of moso‐bamboo between different forest stands based on Tukey's LSD test. GP, Gram‐positive bacteria; ActinoB, Actinobacteria; GN, Gram‐negative bacteria; MethanoB, Methanotrophic bacteria; Fungi, NonAM/ECM fungi; Bamf, bamboo forest stand; Conf, coniferous forests stand; Brof, broadleaf forest stand. Significant differences are indicated by different letters (*p* < .05).

The relative proportion of AB in bamboo rhizosphere in the broadleaf forest stand was significantly lower than in the pure bamboo forest stand, while that in the coniferous forest stand was not significantly different from the other two forests (Figure 4). Besides, the relative proportion of nonmycorrhizal fungi in bamboo rhizosphere in the broadleaf forest stand was higher than in the pure bamboo forest stand, while that in the coniferous forest stand was not significantly different from the other two forests (Figure 4). The major rhizospheric microbial groups of moso‐bamboo were not significant among the three types (Figure 3), as the similar results of relative proportions of bamboo rhizospheric GP, GN, AMF, MB, and ECMF (Figure 4).

### 3.4 Bamboo root morphology

**FIGURE 5:**
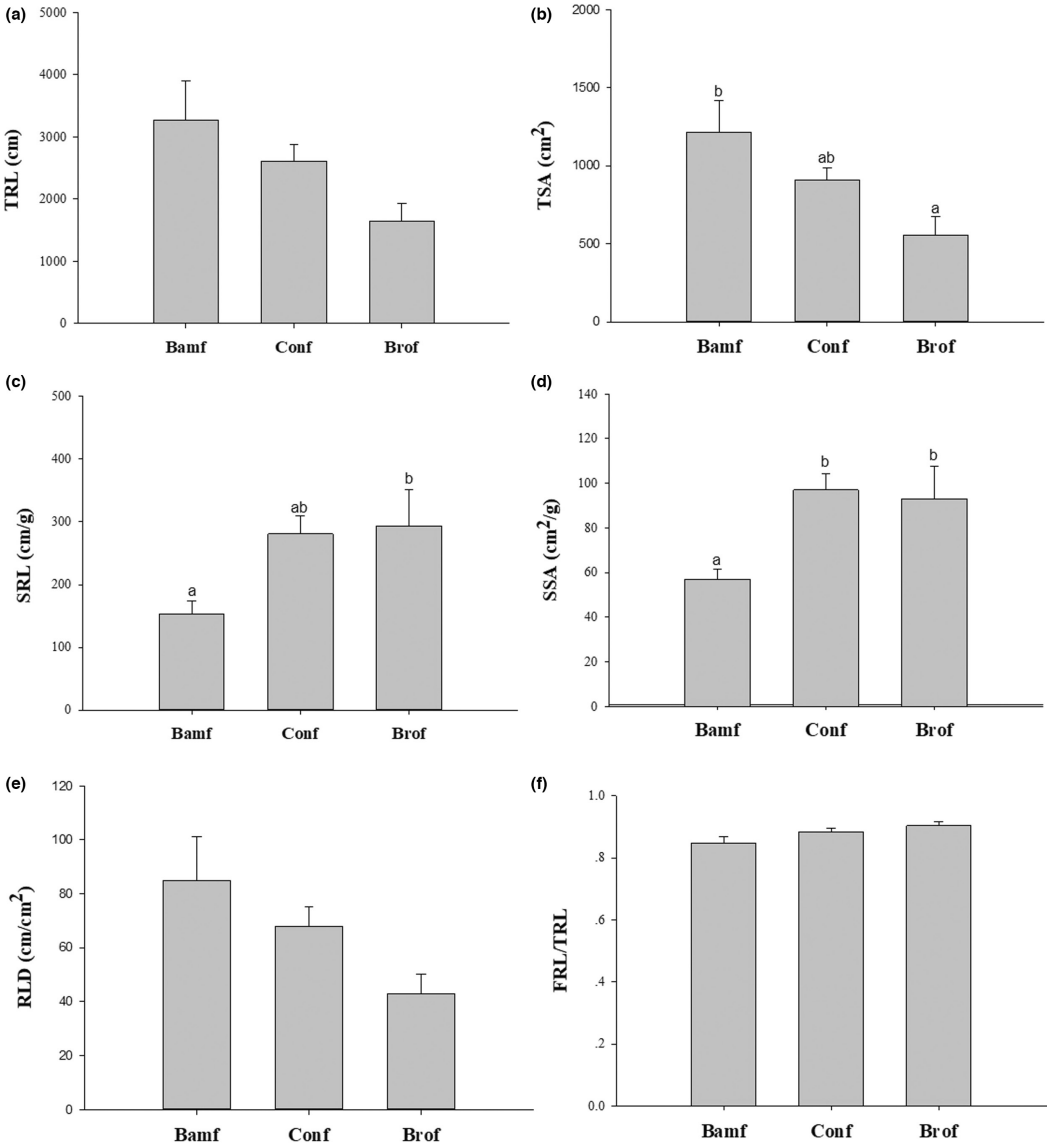
Differences in the root morphology of moso‐bamboo between different forest stands based on Tukey's LSD test. Bamf, bamboo forest stand; Brof, broadleaved forest stand; Conf, coniferous forests stand; RLD, root length density; SRL, specific root length; SSA, specific surface area; TRL, total root length; TSA, total surface area. Significant differences are indicated by different letters (*p* < .05).

Root surface area of the broadleaf forest stand was significantly smaller than that of the bamboo forest stand (Figure 5b). Specific root length in the broadleaf forest stand was significantly longer than that of the conifer and bamboo forest stands (Figure 5c). Specific root surface area in the broadleaf forest stand was smaller than that in the coniferous forest stand and was significantly larger than that in the bamboo forest stand, while it was also significantly larger in the coniferous versus bamboo forest stand (Figure 5d). Root biomass in the broadleaf and coniferous forest stands was much less than that in the bamboo forest stand (Table S3).

### 3.5 The partial least square‐path model of correlation among soil total phosphorus, bamboo rhizosphere microbial community, and bamboo root morphology

**FIGURE 6:**
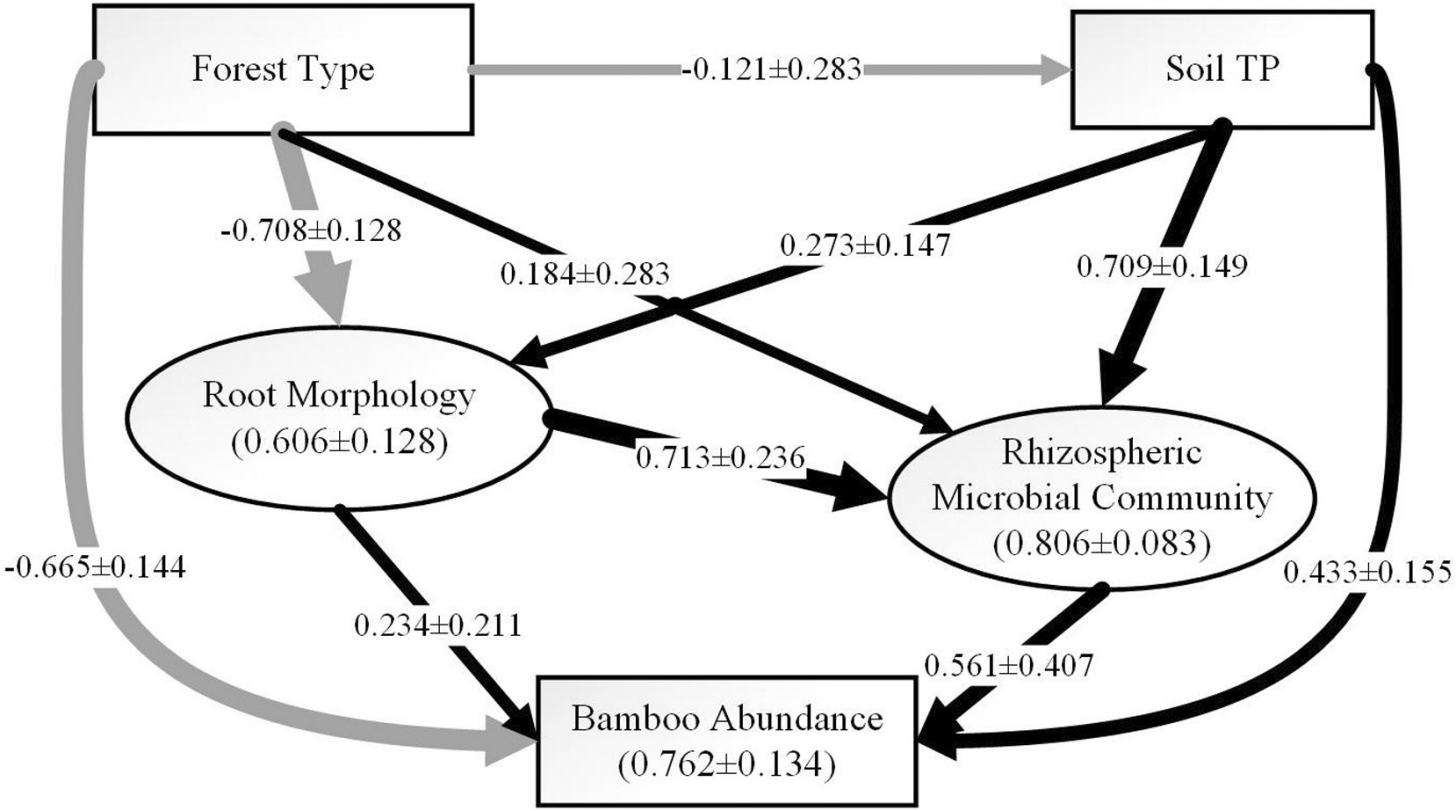
Partial least square path model of correlations among soil TP, root morphology, and rhizospheric microbial community in moso‐bamboo invasion. Path coefficient and standard error are shown. *R* ^2^ values with SE are shown for all endogenous latent variables. Black solid lines and arrows denote facilitation. Light gray lines denote inhibition. Thicker lines indicate stronger correlations. See indicator loadings and path coefficients in Tables S1 and S2, respectively.

According to the PLS‐path model, soil TP did not have a much strong direct effect on bamboo abundance (coefficient = 0.433), but there was a very strong correlation between the soil P content and the composition of rhizosphere microbial community (coefficient = 0.709), indicating a strong indirect effect of soil P on bamboo abundance through altering rhizosphere microbial community. Root morphological traits, specific root surface, total root length, and average diameter, were negatively correlated with bamboo abundance, while they were positively correlated with rhizosphere microbial community. The correlations between forest type and rhizosphere microbe, soil TP and root morphology were weak (Figure 6).

## 4 DISCUSSION

According to our study, the below‐ground performance of moso‐bamboo expanding into neighboring forests could be partially explained by alterations in root morphology and rhizosphere microbial community (Curt et al., 2005; Liu et al., 2019; Wang, Sasaki, et al., 2016). In particular, soil P content may play a key role in driving such below‐ground changes. Although both the broadleaf and coniferous forest stands were more relatively P limited than pure bamboo forests, moso‐bamboo may invade these two types of forests through different mechanisms. Alterations in root morphology may be the key to successful invasion into broadleaf forests, whereas alterations in bamboo‐microbe interaction may be the key to successful invasion into coniferous forests.

### 4.1 Rhizosphere microbe of moso‐bamboo in different forests

According to path analysis, soil P content was strongly correlated with rhizosphere microbial community; and a high P content in soil reduced AMF abundance in soil, which is consistent with previous studies (Smith & Read, 2008). Root AMF infection rate of moso‐bamboo in the coniferous stand was significantly higher than that in the broadleaf stand (Figure 2). It could be explained that host plants can alleviate soil P limitation with the help of AMF (Bolan, 1991; George et al., 2008; Phillips et al., 2013), indicating that the higher AMF infection rate in bamboo roots in the coniferous compared with broadleaf stand may be an adaptation to stronger P limitation in the former. Similarly, Qin et al. (2017) reported that the symbiotic relationship between moso‐bamboo and mycorrhizal fungi (AMF, ECMF) might change at different stages of expansion to adapt to the changes in soil resources. But the ECMF infection rate of moso‐bamboo in our study was too low (Figure 2a), probably the role of ECMF in moso‐bamboo expansion was not effective as AMF with soil P limitation (Phillips et al., 2013).

In addition to AMF, nonmycorrhizal fungi may also play an important role in moso‐bamboo expansion. The differences in bamboo rhizosphere microbial community across different forest types could be mainly attributed to the changes in the relative abundance of nonmycorrhizal fungi. According to the vegetation data (Appendix S1), there was a negative correlation between bamboo abundance and nonmycorrhizal fungi bamboo relative proportions. The greater proportion of nonmycorrhizal fungi may result from greater microbial diversity in broadleaf compared with coniferous and pure bamboo forests, which may correspond with a greater proportion of pathogenic fungi that have the capacity to prevent moso‐bamboo invasion (Maron et al., 2011; van der Putten, 2000).

Besides nonmycorrhizal fungi, the relative abundance of actinomycetes in bamboo rhizosphere was also significantly different between broadleaf forest and pure bamboo forest. Because actinomycetes had been found to prevent infection by fungal pathogens (Goodfellow & Williams, 1983), there might be an abundance trade‐off between actinomycete and nonmycorrhizal fungi in bamboo rhizosphere, resulting in a smaller relative abundance of actinomycete and a larger relative abundance of nonmycorrhizal fungi in the broadleaf compared with pure bamboo forest stand.

In addition to competing with fungal pathogens, actinomycetes may facilitate litter decomposition in bamboo forests (Goodfellow & Williams, 1983), which may explain why actinomycetes are usually higher proportion in bamboo forests (Hua et al., 2011; Li et al., 2020; Tu et al., 2014). However, how moso‐bamboo manages to increase actinomycete abundance warrants further study.

### 4.2 Root morphology of moso‐bamboo in different forests

Specific root length was significantly longer in broadleaf compared with the bamboo forest stand (Figure 5c), potentially facilitating P uptake in response to soil P limitation., while specific root surface area was significantly larger in the coniferous and broadleaf compared with bamboo forest stand (Figure 5d), potentially in the coniferous stand allowing more AMF association in response to soil P limitation (Figure 2b), but in the broadleaf stand absorbing more P directly by specific root surface area increasing.

Although our results showed that moso‐bamboo was able to produce more roots in pure bamboo forests compared with broadleaf or coniferous forests (Table S3), previous studies suggest that increasing root spatial configuration may be a more efficient way to cope with resource limitation than simply increasing root biomass (Bauhus & Messier, 1999; Bloom et al., 1985; Fitter et al., 2002; Leuschner et al., 2004). Specific root length and specific root surface area are important root morphological traits (Bolte & Villanueva, 2005). The increased specific root length and specific root surface area in the broadleaf versus bamboo forest stand indicates an efficient adaptation to soil P limitation in the former.

In addition, an increase in specific root length is also believed to be beneficial for root competitiveness (Curt et al., 2005; Fujii & Kasuya, 2017; Liu et al., 2013; Metcalfe et al., 2008), which has also been previously reported in moso‐bamboo invasion (Liu et al., 2013).

### 4.3 Different underground mechanisms for moso‐bamboo expanding into broadleaf and coniferous forests

Despite more and more attention on bamboo invasion, most previous studies on this topic have only focused on invasion into either broadleaf or coniferous forests (Cai et al., 2019; Fukushima et al., 2015; Laplace et al., 2017; Shinohara & Otsuki, 2015; Wang, Sasaki, et al., 2016; Xu et al., 2020; Zhang & Xue, 2018). For the few studies that simultaneously compared the invasions into different types of forests (Liu et al., 2019), they found that the invasion of moso‐bamboo changed soil and litter properties differently between the two types of forests. Interestingly, although moso‐bamboo was exposed to stronger soil P limitation in both the broadleaf and coniferous forest stands compared with the pure bamboo forest stand, it coped with P limitation through very different strategies (Wu et al., 2018).

For the expansion into broadleaf forests, moso‐bamboo may have altered its root morphology to increase direct P uptake and root competitiveness. Because of the higher ratio of nonmycorrhizal fungi in the rhizosphere communities (Figure 4), moso‐bamboo would suffer a greater risk of infection by the pathogen, so moso‐bamboo may strengthen competitiveness in root morphology (Figure 5) to respond to the soil P limited and unfriendly rhizosphere communities In contrast, for the expansion into coniferous forests, moso‐bamboo may have enhanced its association with soil microbes, especially AMF(Figure 2) to increase indirect P uptake and reduce microbe infection to respond the situation with strongest P limited among three forest types. Our study is among the first to reveal different mechanisms for moso‐bamboo invasion into forests with different dominant species.

## AUTHOR CONTRIBUTIONS

**Jingyu Liu:** Formal analysis (supporting); writing – original draft (equal). **Huixuan Liao:** Project administration (supporting); supervision (supporting); writing – review and editing (equal). **Minghua Fan:** Formal analysis (lead); investigation (equal). **Ting Zhou:** Funding acquisition (lead); project administration (lead); supervision (supporting). **Shaolin Peng:** Funding acquisition (supporting); supervision (lead).

## FUNDING INFORMATION

This project was funded by Fundo dos Pandas, Guangdong Basic and Applied Basic Research Foundation (2022A15150109312020A1515011265), Guangdong Provincial Construction Project of Agricultural Science and Technology Innovation and Extension System (2023KJ360) and Zhang Hongda Science Foundation and Fu Jia‐Rui Scholarship of Sun Yat‐sen University.

## Supporting information


Data S1.

## Data Availability

The dataset would be available in the Dryad repository, https://doi.org/10.5061/dryad.kprr4xh95.

## References

[ece310153-bib-0001] Bauhus, J. , & Messier, C. (1999). Soil exploitation strategies of fine roots in different tree species of the southern boreal forest of eastern Canada. Canadian Journal of Forest Research, 29(2), 260–273. 10.1139/cjfr-29-2-260

[ece310153-bib-0002] Bloom, A. J. , Chapin, F. S. , & Mooney, H. A. (1985). Resource limitation in plants‐an economic analogy. Annual Review of Ecology and Systematics, 16(1), 363–392. 10.1146/annurev.es.16.110185.002051

[ece310153-bib-0003] Bolan, N. S. (1991). A critical review on the role of mycorrhizal fungi in the uptake of phosphorus by plants. Plant and Soil, 134(2), 189–207. 10.1007/bf00012037

[ece310153-bib-0004] Bolte, A. , & Villanueva, I. (2005). Interspecific competition impacts on the morphology and distribution of fine roots in European beech (Fagus sylvatica L.) and Norway spruce (Picea abies (L.) karst.). European Journal of Forest Research, 125(1), 15–26. 10.1007/s10342-005-0075-5

[ece310153-bib-0005] Cai, C.‐J. , Fan, S.‐H. , Liu, X.‐Z. , & Liu, G.‐ l. (2019). Fine root adaptation strategy of moso bamboo during its expansion into Chinese fir forest. Shengtaixue Zazhi, 38(4), 967–972. 10.13292/j.1000-4890.201904.019

[ece310153-bib-0006] Curt, T. , Coll, L. , Prvosto, B. , Balandier, P. , & Kunstler, G. (2005). Plasticity in growth, biomass allocation and root morphology in beech seedlings as induced by irradiance and herbaceous competition. Annals of Forest Science, 62(1), 51–60. 10.1051/forest:2004092

[ece310153-bib-0007] Fitter, A. , Williamson, L. , Linkohr, B. , & Leyser, O. (2002). Root system architecture determines fitness in an Arabidopsis mutant in competition for immobile phosphate ions but not for nitrate ions. Proceeding of the Royal Society Biological Science, 269(1504), 2017–2022. 10.1098/rspb.2002.2120 PMC1691122 12396500

[ece310153-bib-0008] Frostegård, Å. , Tunlid, A. , & Bååth, E. (2011). Use and misuse of PLFA measurements in soils. Soil Biology and Biochemistry, 43(8), 1621–1625. 10.1016/j.soilbio.2010.11.021

[ece310153-bib-0009] Fujii, S. , & Kasuya, N. (2017). Fine root biomass and morphology of Pinus densiflora under competitive stress by Chamaecyparis obtusa. Journal of Forest Research, 13(3), 185–189. 10.1007/s10310-008-0063-y

[ece310153-bib-0010] Fukushima, K. , Usui, N. , Ogawa, R. , & Tokuchi, N. (2015). Impacts of moso bamboo (Phyllostachys pubescens) invasion on dry matter and carbon and nitrogen stocks in a broad‐leaved secondary forest located in Kyoto, western Japan. Plant Species Biology, 30(2), 81–95. 10.1111/1442-1984.12066

[ece310153-bib-0011] George, E. , Marschner, H. , & Jakobsen, I. (2008). Role of arbuscular mycorrhizal fungi in uptake of phosphorus and nitrogen from soil. Critical Reviews in Biotechnology, 15(3–4), 257–270. 10.3109/07388559509147412

[ece310153-bib-0012] Goodfellow, M. , & Williams, S. T. (1983). Ecology of actinomycetes. Annual Review of Microbiology, 37, 189–216. 10.1146/annurev.mi.37.100183.001201 6357051

[ece310153-bib-0013] Griscom, B. W. , & Ashton, P. M. S. (2003). Bamboo control of forest succession: Guadua sarcocarpa in southeastern Peru. Forest Ecology and Management, 175(1–3), 445–454. 10.1016/s0378-1127(02)00214-1

[ece310153-bib-0014] Hua, L. , Chen, Y. , Wu, W. , & Ma, H. (2011). Microorganism communities and chemical characteristics in sludge‐bamboo charcoal composting system. Environmental Technology, 32(5–6), 663–672. 10.1080/09593330.2010.510534 21877547

[ece310153-bib-0015] Komatsu, H. , Onozawa, Y. , Kume, T. , Tsuruta, K. , Kumagai, T. O. , Shinohara, Y. , & Otsuki, K. (2010). Stand‐scale transpiration estimates in a Moso bamboo forest: II. Comparison with coniferous forests. Forest Ecology and Management, 260(8), 1295–1302. 10.1016/j.foreco.2010.06.040

[ece310153-bib-0016] Laplace, S. , Komatsu, H. , Tseng, H. , & Kume, T. (2017). Difference between the transpiration rates of Moso bamboo (*Phyllostachys pubescens*) and Japanese cedar (*Cryptomeria japonica*) forests in a subtropical climate in Taiwan. Ecological Research, 32(6), 835–843. 10.1007/s11284-017-1512-x

[ece310153-bib-0017] Larpkern, P. , Moe, S. R. , & Totland, O. (2011). Bamboo dominance reduces tree regeneration in a disturbed tropical forest. Oecologia, 165(1), 161–168. 10.1007/s00442-010-1707-0 20607296

[ece310153-bib-0018] Leuschner, C. , Hertel, D. , Schmid, I. , Koch, O. , Muhs, A. , & Hölscher, D. (2004). Stand fine root biomass and fine root morphology in old‐growth beech forests as a function of precipitation and soil fertility. Plant and Soil, 258(1), 43–56. 10.1023/b:Plso.0000016508.20173.80

[ece310153-bib-0019] Li, L. , Li, N. , Lu, D. , & Chen, Y. (2019). Mapping Moso bamboo forest and its on‐year and off‐year distribution in a subtropical region using time‐series Sentinel‐2 and Landsat 8 data. Remote Sensing of Environment, 231, 111265. 10.1016/j.rse.2019.111265

[ece310153-bib-0020] Li, W. , Tian, X. , Sheng, H. , Ekawati, D. , Zhou, Y. , & Zhang, R. (2020). Response of bacterial compositions to soil biochemical properties under mulching‐intensive management in a Phyllostachys edulis forest. Applied Soil Ecology, 150, 103436. 10.1016/j.apsoil.2019.103436 PMC6926336 31890811

[ece310153-bib-0021] Lin, Y.‐T. , Tang, S.‐L. , Pai, C.‐W. , Whitman, W. B. , Coleman, D. C. , & Chiu, C.‐Y. (2014). Changes in the soil bacterial communities in a cedar plantation invaded by Moso bamboo. Microbial Ecology, 67(2), 421–429. 10.1007/s00248-013-0291-3 24072077

[ece310153-bib-0022] Liu, J. , Yang, Q.‐P. , Song, Q.‐N. , Yu, D.‐K. , Yang, G.‐Y. , Qi, H.‐Y. , & Shi, J.‐M. (2013). Strategy of fine root expansion of Phyllostachys pubescens population into evergreen broad‐leaved forest. Chinese Journal of Plant Ecology, 37(3), 230–238. 10.3724/sp.J.1258.2013.00023

[ece310153-bib-0023] Liu, X. , Siemann, E. , Cui, C. , Liu, Y. , Guo, X. , & Zhang, L. (2019). Moso bamboo (Phyllostachys edulis) invasion effects on litter, soil and microbial PLFA characteristics depend on sites and invaded forests. Plant and Soil, 438(1–2), 85–99. 10.1007/s11104-019-04010-3

[ece310153-bib-0024] Maron, J. L. , Marler, M. , Klironomos, J. N. , & Cleveland, C. C. (2011). Soil fungal pathogens and the relationship between plant diversity and productivity. Ecology Letters, 14(1), 36–41. 10.1111/j.1461-0248.2010.01547.x 21073641

[ece310153-bib-0025] Mcgonigle, G. T. , Miller, M. H. , Evans, D. G. , Fairchild, G. L. , & Swan, J. A. (1990). A new method which gives an objective measure of colonization of roots by vesicular‐arbuscular mycorrhizal fungi. New Phytologist, 115(3), 495–501. 10.1111/j.1469-8137.1990.tb00476.x 33874272

[ece310153-bib-0026] Metcalfe, D. B. , Meir, P. , Aragão, L. E. O. C. , da Costa, A. C. L. , Braga, A. P. , Gonçalves, P. H. L. , de Athaydes Silva Junior, J. , de Almeida, S. S. , Dawson, L. A. , Malhi, Y. , & Williams, M. (2008). The effects of water availability on root growth and morphology in an Amazon rainforest. Plant and Soil, 311(1–2), 189–199. 10.1007/s11104-008-9670-9

[ece310153-bib-0027] Okutomi, K. , Shinoda, S. , & Fukuda, H. (1996). Causal analysis of the invasion of broad‐leaved forest by bamboo in Japan. Journal of Vegetation Science, 7(5), 723–728. 10.2307/3236383

[ece310153-bib-0028] Phillips, R. P. , Brzostek, E. , & Midgley, M. G. (2013). The mycorrhizal‐associated nutrient economy: A new framework for predicting carbon‐nutrient couplings in temperate forests. New Phytologist, 199(1), 41–51. 10.1111/nph.12221 23713553

[ece310153-bib-0029] Qin, H. , Niu, L. , Wu, Q. , Chen, J. , Li, Y. , Liang, C. , Xu, Q. , Fuhrmann, J. J. , & Shen, Y. (2017). Bamboo forest expansion increases soil organic carbon through its effect on soil arbuscular mycorrhizal fungal community and abundance. Plant and Soil, 420(1–2), 407–421. 10.1007/s11104-017-3415-6

[ece310153-bib-0030] Shinohara, Y. , & Otsuki, K. (2015). Comparisons of soil‐water content between a Moso bamboo (Phyllostachys pubescens) forest and an evergreen broadleaved forest in western Japan. Plant Species Biology, 30(2), 96–103. 10.1111/1442-1984.12076

[ece310153-bib-0031] Smith, S. E. , & Read, D. F. (2008). Mycorrhizal symbiosis (3rd ed.). Academic Press.

[ece310153-bib-0032] Tripathi, S. K. , Sumida, A. , Ono, K. , Shibata, H. , Uemura, S. , Takahashi, K. , & Hara, T. (2005). The effects of understorey dwarf bamboo (Sasa kurilensis) removal on soil fertility in a Betula ermanii forest of northern Japan. Ecological Research, 21(2), 315–320. 10.1007/s11284-005-0119-9

[ece310153-bib-0033] Tu, Z. , Chen, L. , Yu, X. , & Zheng, Y. (2014). Rhizosphere soil enzymatic and microbial activities in bamboo forests in southeastern China. Soil Science and Plant Nutrition, 60(2), 134–144. 10.1080/00380768.2014.882219

[ece310153-bib-0034] van der Putten, W. H. (2000). Pathogen‐driven forest diversity. Nature, 404(6775), 232–233. 10.1038/35005188 10749191

[ece310153-bib-0035] Vestal, J. R. , & White, D. C. (1989). Lipid analysis in microbial ecology. Bioscience, 39(8), 535–541. 10.2307/1310976 11542183

[ece310153-bib-0036] Wang, D. , Yu, S. B. , & Zhang, Y. L. (2017). Changes and influencing factors of soil carbon in evergreen broadleaved forest by phyllostachys pubescens in Jiangxi province, South China. Journal of Tropical Forest Science, 29(1), 37–43.

[ece310153-bib-0037] Wang, H.‐C. , Tian, G. , & Chiu, C.‐Y. (2016). Invasion of moso bamboo into a Japanese cedar plantation affects the chemical composition and humification of soil organic matter. Scientific Reports, 6, 1–6. 10.1038/srep32211 27558833 PMC4997307

[ece310153-bib-0038] Wang, X. , Sasaki, A. , Toda, M. , & Nakatsubo, T. (2016). Changes in soil microbial community and activity in warm temperate forests invaded by moso bamboo (Phyllostachys pubescens). Journal of Forest Research, 21(5), 235–243. 10.1007/s10310-016-0533-6

[ece310153-bib-0039] Wang, Y. , Bai, S. , Binkley, D. , Zhou, G. , & Fang, F. (2016). The independence of clonal shoot's growth from light availability supports moso bamboo invasion of closed‐canopy forest. Forest Ecology and Management, 368, 105–110. 10.1016/j.foreco.2016.02.037

[ece310153-bib-0040] Wu, C. , Mo, Q. , Wang, H. , Zhang, Z. , Huang, G. , Ye, Q. , Zou, Q. , Kong, F. , Liu, Y. , & Geoff Wang, G. (2018). Moso bamboo (*Phyllostachys edulis* (Carriere) J. Houzeau) invasion affects soil phosphorus dynamics in adjacent coniferous forests in subtropical China. Annals of Forest Science, 75(1), 24. 10.1007/s13595-018-0703-0

[ece310153-bib-0041] Xu, Q.‐F. , Liang, C.‐F. , Chen, J.‐H. , Li, Y.‐C. , Qin, H. , & Fuhrmann, J. J. (2020). Rapid bamboo invasion (expansion) and its effects on biodiversity and soil processes. Global Ecology and Conservation, 21, e00787. 10.1016/j.gecco.2019.e00787

[ece310153-bib-0042] Zelles, L. (1999). Fatty acid patterns of phospholipids and lipopolysaccharides in the characterisation of microbial communities in soil: A review. Biology and Fertility of Soils, 29(2), 111–129. 10.1007/s003740050533

[ece310153-bib-0043] Zhang, H. , & Xue, J. (2018). Spatial pattern and competitive relationships of Moso bamboo in a native subtropical rainforest community. Forests, 9(12), 774. 10.3390/f9120774

