# Supplementary material for: Comparison of root morphology and rhizosphere microbial communities form moso‐bamboo in different forest types
Source: Ecol Evol. 2023 Jun 6;13(6):e10153. doi: 10.1002/ece3.10153 (PMC10245033; doi:10.1002/ece3.10153)
Supplement: Supplementary file 1 — Data S1. [file ECE3-13-e10153-s001.zip › ECE3_10153_Appendix S1.docx]

**Appendix S1**

Vegetation characteristics of three Forest types in the Nankunshan Natural Reserve.

Relative Abundance = abundance of one species/abundance of all species

Relative frequency = frequency of one species/ frequency of all species

Relative Prominence = Prominence of one species/ Prominence of all species

Prominence = Sum of stand basal area areas of all individuals

Importance Value = (Relative Abundance + Relative frequency + Relative Prominence) *100

The IVs of species were bolded, which were the dominating species of the forest types.

| Forest Type | Specie | Relative Abundance | Relative frequency | Relative Prominence | Importance Value(IV) |
| --- | --- | --- | --- | --- | --- |
| Bamboo Forest | *Phyllostachys edulis* | 0.93 | 0.43 | 0.80 | **215.80** |
| Bamboo Forest | *Cunninghamia lanceolata* | 0.05 | 0.36 | 0.20 | **61.26** |
| Bamboo Forest | *Schima superba* | 0.01 | 0.14 | NA | NA |
| Bamboo Forest | *Heteropanax brevipedicellatus* | 0.00 | 0.07 | 0.00 | 7.59 |
| Broadleaf Forest | *Phyllostachys edulis* | 0.37 | 0.08 | 0.23 | **68.89** |
| Broadleaf Forest | *Cunninghamia lanceolata* | 0.08 | 0.07 | 0.23 | **37.54** |
| Broadleaf Forest | *Castanopsis carlesii* | 0.05 | 0.06 | 0.21 | **31.75** |
| Broadleaf Forest | *Bambusa textilis* | 0.11 | 0.06 | 0.03 | 20.33 |
| Broadleaf Forest | *Diospyros morrisiana* | 0.03 | 0.06 | 0.04 | 12.52 |
| Broadleaf Forest | *Castanopsis fabri* | 0.03 | 0.06 | 0.02 | 10.82 |
| Broadleaf Forest | *Abarema lucida* | 0.01 | 0.04 | 0.02 | 7.01 |
| Broadleaf Forest | *Itea chinensis* | 0.01 | 0.04 | 0.00 | 5.56 |
| Broadleaf Forest | *Rhodoleia championii* | 0.03 | 0.03 | 0.07 | 13.15 |
| Broadleaf Forest | *Cinnamomum parthenoxylon* | 0.01 | 0.03 | 0.04 | 7.95 |
| Broadleaf Forest | *Symplocos lancifolia* | 0.03 | 0.03 | 0.01 | 7.30 |
| Broadleaf Forest | *Aidia cochinchinensis* | 0.03 | 0.03 | 0.01 | 6.53 |
| Broadleaf Forest | *Machilus chinensis* | 0.02 | 0.03 | 0.00 | 5.27 |
| Broadleaf Forest | *Ormosia semicastrata* | 0.01 | 0.03 | 0.01 | 4.95 |
| Broadleaf Forest | *Ternstroemia gymnanthera* | 0.01 | 0.03 | 0.00 | 4.18 |
| Broadleaf Forest | *Diplospora dubia* | 0.01 | 0.03 | 0.00 | 4.11 |
| Broadleaf Forest | *Lithocarpus calophyllus* | 0.03 | 0.01 | 0.02 | 6.06 |
| Broadleaf Forest | *Machilus velutina* | 0.02 | 0.01 | 0.00 | 3.56 |
| Broadleaf Forest | *Machilus breviflora* | 0.01 | 0.01 | 0.01 | 3.21 |
| Broadleaf Forest | *Neolitsea chuii* | 0.01 | 0.01 | 0.00 | 3.01 |
| Broadleaf Forest | *Elaeocarpus sylvestris* | 0.01 | 0.01 | 0.01 | 2.73 |
| Broadleaf Forest | *Engelhardtia roxburghiana* | 0.01 | 0.01 | 0.00 | 2.47 |
| Broadleaf Forest | *Syzygium austrosinense* | 0.01 | 0.01 | 0.00 | 2.41 |
| Broadleaf Forest | *Xanthophyllum hainanense* | 0.00 | 0.01 | 0.01 | 2.32 |
| Broadleaf Forest | *Adinandra millettii* | 0.01 | 0.01 | 0.00 | 2.23 |
| Broadleaf Forest | *Ehretia longiflora* | 0.00 | 0.01 | 0.00 | 2.15 |
| Broadleaf Forest | *Reevesia thyrsoidea* | 0.00 | 0.01 | 0.00 | 2.10 |
| Broadleaf Forest | *Ilex ficoidea* | 0.00 | 0.01 | 0.00 | 2.01 |
| Broadleaf Forest | *Ficus variolosa* | 0.00 | 0.01 | 0.00 | 1.87 |
| Broadleaf Forest | *Illicium lanceolatum* | 0.00 | 0.01 | 0.00 | 1.81 |
| Broadleaf Forest | *Michelia skinneriana* | 0.00 | 0.01 | 0.00 | 1.80 |
| Broadleaf Forest | *Artocarpus styracifolius* | 0.00 | 0.01 | 0.00 | 1.79 |
| Broadleaf Forest | *Eurya loquaiana* | 0.00 | 0.01 | 0.00 | 1.78 |
| Broadleaf Forest | *Camellia oleifera* | 0.00 | 0.01 | 0.00 | 1.78 |
| Broadleaf Forest | *Schima superba* | 0.00 | 0.01 | 0.00 | 1.78 |
| Broadleaf Forest | *Michelia foveolata* | 0.00 | 0.01 | 0.00 | 1.76 |
| Broadleaf Forest | *Camellia cordifolia* | 0.00 | 0.01 | 0.00 | 1.75 |
| Broadleaf Forest | *Croton lachnocarpus* | 0.00 | 0.01 | 0.00 | 1.75 |
| Coniferous Forest | *Phyllostachys edulis* | 0.79 | 0.26 | 0.53 | **157.54** |
| Coniferous Forest | *Cunninghamia lanceolata* | 0.09 | 0.22 | 0.28 | **58.61** |
| Coniferous Forest | *Schima superba* | 0.02 | 0.09 | 0.08 | 18.59 |
| Coniferous Forest | *Castanopsis fabri* | 0.02 | 0.09 | 0.03 | 13.61 |
| Coniferous Forest | *Litsea cubeba* | 0.01 | 0.09 | 0.00 | 9.48 |
| Coniferous Forest | *Bambusa textilis* | 0.05 | 0.04 | 0.02 | 11.13 |
| Coniferous Forest | *Rhodoleia championii* | 0.01 | 0.04 | 0.05 | 9.72 |
| Coniferous Forest | *Machilus breviflora* | 0.01 | 0.04 | 0.00 | 5.82 |
| Coniferous Forest | *Diospyros morrisiana* | 0.00 | 0.04 | 0.01 | 5.44 |
| Coniferous Forest | *Lithocarpus calophyllus* | 0.00 | 0.04 | 0.01 | 5.30 |
| Coniferous Forest | *Engelhardtia fenzelii* | 0.00 | 0.04 | 0.00 | 4.74 |
