# Supplementary material for: Comparison of root morphology and rhizosphere microbial communities form moso‐bamboo in different forest types
Source: Ecol Evol. 2023 Jun 6;13(6):e10153. doi: 10.1002/ece3.10153 (PMC10245033; doi:10.1002/ece3.10153)

**Table S1** Cross loadings of PLS path models.

|  | **ForestType** | **Soil TP** | **Root** | **Microbe** | **Abundance** |
| --- | --- | --- | --- | --- | --- |
| **ForestType** | 1 | -0.126 | -0.704 | -0.185 | -0.672 |
| **Soil TP** | -0.126 | 1 | 0.365 | 0.737 | 0.507 |
| **Root morphlogy** | -0.704 | 0.365 | 1 | 0.633 | 0.682 |
| **Microbial community** | -0.185 | 0.737 | 0.633 | 1 | 0.607 |
| **Bamboo Abundance** | -0.672 | 0.507 | 0.682 | 0.607 | 1 |

**Table S2** Bootstrap validation of the path coefficients in PLS path models.

| **Path** | **Original** | **Mean** | **SE** | **perc.025** | **perc.975** |
| --- | --- | --- | --- | --- | --- |
| **ForestType -> Soil TP** | -0.126 | -0.1209 | 0.283 | -0.6483 | 0.433 |
| **ForestType -> Root morphlogy** | -0.669 | -0.6847 | 0.126 | -0.896 | -0.404 |
| **ForestType -> Microbial community** | 0.381 | 0.3824 | 0.193 | 0.0299 | 0.818 |
| **ForestType -> Bamboo Abundance** | -0.657 | -0.6388 | 0.252 | -1.1386 | -0.176 |
| **Soil TP-> Root morphlogy** | 0.28 | 0.2728 | 0.147 | -0.0216 | 0.545 |
| **Soil TP -> Microbial community** | 0.526 | 0.5227 | 0.145 | 0.229 | 0.807 |
| **Soil TP-> Bamboo Abundance** | 0.118 | 0.0685 | 0.302 | -0.5958 | 0.594 |
| **Root morphlogy -> Microbial community** | 0.709 | 0.7129 | 0.236 | 0.2684 | 1.173 |
| **Root morphlogy -> Bamboo Abundance** | -0.125 | -0.1665 | 0.372 | -0.9682 | 0.409 |
| **Microbial community -> Bamboo Abundance** | 0.477 | 0.5611 | 0.407 | -0.1554 | 1.438 |

**Table S3.** Root morphological trait of moso bamboo in different forest stands. TRL: total rootlength; TSA: total surface area; SRL: specific root length; SSA: specific surface area; RLD: root length density; Bamf: bamboo forests stand; Conf: coniferous forests stand; Brof: broadleaved forests stand. Mean and standard error are shown. Significant differences are indicated by different letters (P < 0.05)

| **Root morphological trait** | **Bamf** | **Conf** | **Brof** |
| --- | --- | --- | --- |
| **TRL(cm)** | 3265.46±629.44 | 2610.45±274.76 | 1648.12±281.27 |
| **TSA(cm^2^)** | 1214.48±201.51 b | 907.96±77.15 ab | 553.15±121.07 a |
| **Root diameter(mm)** | 1.84±0.31 | 1.25±0.20 | 1.16±0.26 |
| **SRL(cm/g)** | 153.46±20.13 a | 280.52±29.47 ab | 294.11±57.71 b |
| **SSR(cm^2^/g)** | 56.86±4.81 a | 96.95±7.48 b | 92.98±14.81 b |
| **Fine root ratio** | 0.85±0.02 | 0.88±0.01 | 0.90±0.01 |
| **RLD(cm/cm^2^)** | 84.89±16.36 | 67.87±7.14 | 42.85±7.31 |
| **Biomass(g/cm^2^)** | 0.58±0.12 b | 0.25±0.02 a | 0.18±0.06 a |

**Figure S1** The map of experimental field forest stands location in Nankunshan Natural Reserve


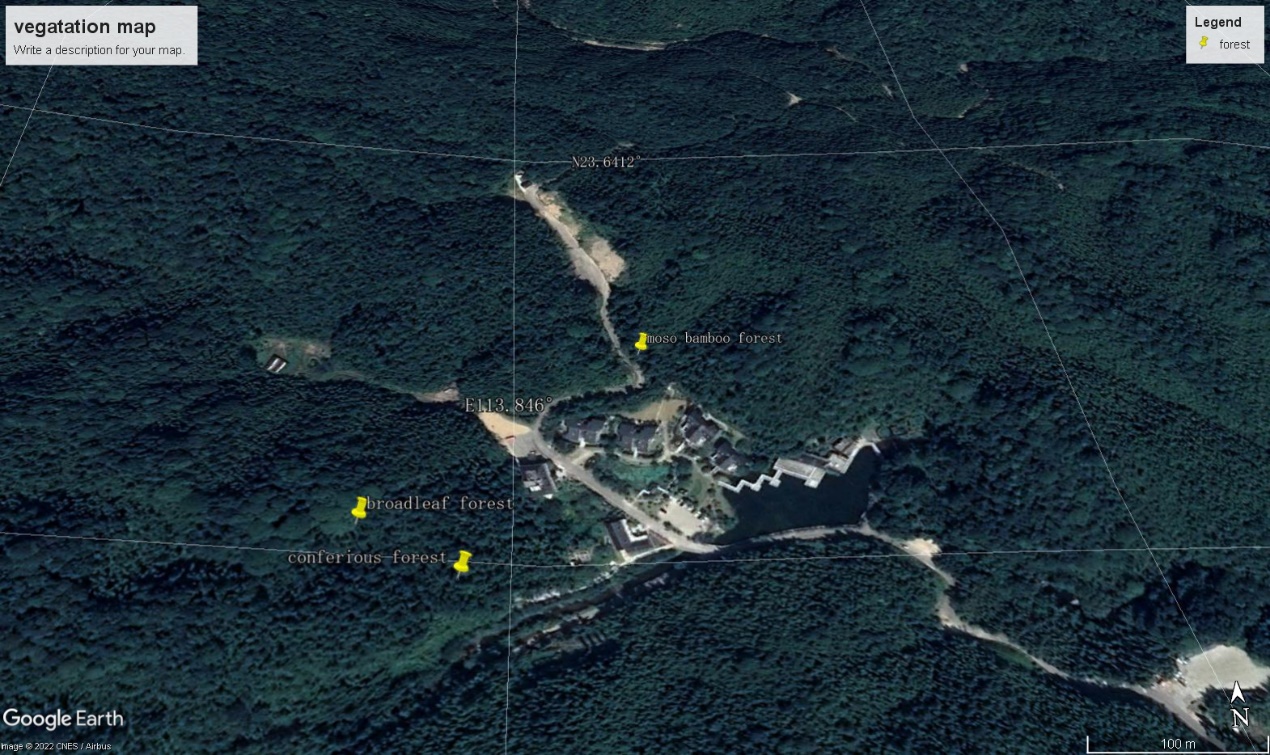

Supplement: Supplementary file 1 — Data S1. [file ECE3-13-e10153-s001.zip › ECE3_10153_Table_S1-3 Figure_S1_SuppInfo.docx]
